# Space Travel: The Radiation and Microgravity Effects on the Cardiovascular System

**DOI:** 10.3390/ijms252111812

**Published:** 2024-11-03

**Authors:** Andrei Alexandru Mircea, Dan Valentin Pistritu, Andra Fortner, Antoanela Tanca, Elisa Anamaria Liehn, Octavian Bucur

**Affiliations:** 1 Faculty of Medicine, Carol Davila University of Medicine and Pharmacy, 020021 Bucharest, Romania; 2 Victor Babes National Institute of Pathology, 050096 Bucharest, Romania; 3 Medical School, Ruprecht-Karls-Universität Heidelberg, 69120 Heidelberg, Germany; 4 Institute for Molecular Medicine, University of Southern Denmark, 5230 Odense, Denmark; 5 Viron Molecular Medicine Institute, Boston, MA 02451, USA; 6 Genomics Research and Development Institute, 020021 Bucharest, Romania

**Keywords:** microgravity, radiation, high atomic number and energy (HZE) particles, endothelial dysfunction

## Abstract

Space flight modulates the functions of the cardiovascular system. The exposure to space conditions can alter the cerebral blood flow, as well as the venous return. Anemia, cardiac output changes, and increased activity of the sympathetic nervous system can also be seen. Understanding cardiac changes prepares astronauts for both better in-flight adaptations and long-term protection against cardiovascular diseases. The heart could undergo radio-degenerative effects when exposed to space radiation, increasing the risk of cardiovascular diseases in the long run. A high frequency of arrhythmias, such as ventricular/atrial premature complexes, have been reported during the Gemini and Apollo missions. Additionally, microgravity can lead to progressive degeneration of the myocytes and muscle atrophy with altered gene expression and calcium handling, along with impaired contractility. This review summarizes the potential cardiovascular effects of spaceflight and prevention measures.

## 1. Introduction

Pushing the boundaries of space exploration is evident with the advent of deep space missions to Mars. It is vital to ensure that such a voyage is sustainable for the human body. Our main objective in this review is to paint the picture of the effects that microgravity and radiation have on the cardiovascular system at both the small scale (i.e., molecular and cellular), as well as the larger scale (i.e., organs/organism level). In order to provide effective countermeasures against the deleterious effects of the spaceflight environment, it is essential to develop a deep understanding of the underlying processes.

The space environment increases the risk of vascular damage, both arterial and venous [1]. In particular, microgravity alters the autonomous nervous system and reduces metabolite clearance. Additionally, space radiation can induce arterial endothelial damage [2]. The venous function is affected through the initial hypovolemic state caused by microgravity and the perivascular space expansion later [3,4].

The second concern regards cardiac function and cardiac monitoring during spaceflight. The cardiovascular system is exposed to various environmental factors that can potentially cause damage. Significant modifiers of the cardiac function are charged particles, particularly high atomic number and energy (HZE) particles [5]. Therefore, in order to assess general cardiac function, a tool to remotely monitor cardiac health in space would be highly beneficial. The rise of wearable devices for measuring heart rate variability (HRV) in the last decade prompts the inquiry as to whether this method is appropriate for space travel [6,7,8]. Spaceflight not only influences HRV, but also ultradian rhythms and other physiological processes, which at least in part are a result of gene expression modifications [9]. Genetic or genomic-based strategies to investigate and monitor gene expression (e.g., in hair follicles) or genomic modifications can help in identifying and monitoring health changes during space travel [10].

All in all, urgent research directions are based on the current gaps in knowledge. We identified at least two, which we discuss in this review. The first is therapy against radiation. The second is prevention against the effects of microgravity.

Astronauts are exposed to a unique and challenging environment during space missions, where microgravity and radiation are two of the most significant concerns with regard to their health. In this review, we have chosen to examine these two stressors individually, focusing on the distinct effects each has on cardiovascular function and structure. By highlighting the separate influences of microgravity and radiation, this study aims to provide a clearer understanding of the mechanisms underlying their cardiovascular impact, though recognizing the limitations of this approach, given the simultaneous exposure astronauts experience.

## 2. How Does Radiation Impact the Cardiovascular System?

### 2.1. Types of Space Radiation

The three major types of ionizing radiation in the space environment are galactic cosmic rays, solar cosmic rays, and charged particles trapped within the Van Allen radiation belts (Table 1) [11,12]. Galactic cosmic rays are a dominant source of space radiation and typically consist of high-energy ions traveling nearly at the speed of light. Of most concern are HZE ions [high (H) atomic number (Z) and energy (E)], which are highly penetrative and damaging to the human body. On Earth, the planet’s atmosphere and magnetic field are shielding it from these particles. However, this protection wanes beyond the low Earth orbit [12]. Solar cosmic rays, which are expulsions of high-energy particles during coronal mass ejections, can significantly add to the injurious radiation effect of constant galactic cosmic rays and increase the risk of radiation-induced cardiovascular disease [13,14]. Currently, the pathophysiology underlying space radiation-induced cardiovascular disease is not completely understood [15]. A recent study showed that a control-matched astronaut cohort did not have an increase in CV mortality but had a significant 2.41-fold increase in cardiovascular disease events [16].

### 2.2. Effects of Radiation on the Heart

It is important to highlight that the extent of any circulatory disease risk associated with doses below 0.5 Gy or below remains especially unclear [17]. Moderate radiation doses, equivalent to those experienced by Atomic Bomb survivors and occupationally exposed workers (0–2 Gy), which are comparable to effective doses for an ISS mission, have also been involved in cardiovascular disease [18,19]. These doses are correlated with elevated risks of hypertension, stroke, and myocardial infarction, particularly among individuals under 40 years old [19].

**Figure 1:**
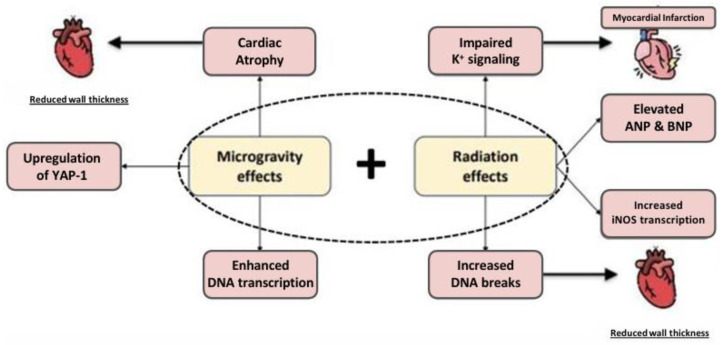
Effects of radiation and microgravity [20,21,22,23,24,25,26,27,28].

Animal studies provide further evidence supporting the deleterious effects of radiation exposure on CV organs. Recent findings have emphasized the significance of myocardial remodeling following radiation-induced cardiac injury and its role in cardiomyocyte damage and myocardial fibrosis. The extent of these effects is dependent on the HZE particle type. For instance, male Long Evans murine models exposed to whole-body protons (250 MeV, 0.5 Gy) and oxygen ions (^16^O, 600 MeV/nucleon, 0.5 Gy) showed a decrease in the thickness of the posterior wall of the left ventricle 3–5 months post-irradiation. However, this change in wall thickness did not alter the cardiac function assessed through echocardiography. The measured parameters included cardiac volume, left ventricular anterior and posterior wall thicknesses, ejection fraction, fractional shortening, stroke volume and cardiac output [20,21]. In addition, proton and oxygen ion exposure only induced cardiac remodeling of the existing collagen, preserving the total collagen content. On the other hand,^56^Fe ion irradiation (0.15 Gy, 1 GeV/nucleon) has been shown to enhance cardiac fibrosis in adult male C57Bl/6NT mice, resulting in acute myocardial ischemic events. More precisely, ^56^Fe ion-irradiated cardiac tissues revealed dysregulations in calcium handling mechanisms through upregulation of Sarcoplasmic Reticulum Ca^2+^ ATPase (SERCA2a) and sodium-calcium exchanger proteins. These changes may account for the decreased left ventricular contractility (dP/dt ratios) seen after one-month post-irradiation, indicating systolic and diastolic dysfunction (Figure 1) [22].

Strong solar particle events may contribute to myocardial infarction events and HRV modifications. These solar particle events cause geomagnetic storms, which are disturbances in the Earth’s magnetic field. In turn, geomagnetic storms exert the aforementioned cardiac effects. Although the potential association between geomagnetic activity and cardiovascular health is still controversial, some studies (Table 2) suggest that high solar activity increases the occurrence of myocardial infarction (MI) and MI mortality [29,30,31]. It has been hypothesized that the reduction in heart rate variability involved in the development of myocardial infarction can be caused by geomagnetic disturbances [29,32,33,34,35]. Notably, very low frequency (VLF) and low frequency (LF) rhythms were found to be decreased, whereas high frequency (HF) has been reported to be either decreased or unchanged [32].

It is unclear whether radiation causes long-term cardiac damage. Radiation has been shown to increase ROS and the risk of cardiovascular disease. However, the long-term effects are far from being demonstrated after the astronauts’ return to Earth [36].

**Table 2:** Overview of human studies investigating the effects of spaceflight on the cardiovascular system.

Study	Institution	Year	Primary Outcome Studied	No. of Subjects	Spaceflight Duration
Longitudinal MRI-visible perivascular space (PVS) changes with long-duration spaceflight [4]	Oregon Health and Science University, USA	2022	Examine how the number and morphology of magnetic resonance imaging-visible PVSs are affected by spaceflight in experienced and inexperienced astronauts	15 astronauts	6 months
Designing a Novel Monitoring Approach for the Effects of Space Travel on Astronauts’ Health [10]	University of Saskatchewan	2023	Gene expression and correlation patterns in pre-flight and in-flight conditions	10 astronauts	Not specified
Long-term Cardiovascular Risk in Astronauts [16]	NASA, USA	2022	Determine the long-term cardiovascular disease risk of astronauts with spaceflight exposure compared with a well-matched cohort	303 humans	Not specified
A comprehensive analysis of incidence of myocardial infarction in Vladikavkaz depending on solar and geomagnetic activity [30] Correlation between Changes in Local Earth’s Magnetic Field and Cases of Acute Myocardial Infarction [31] Synchronization of Human Autonomic Nervous System Rhythms with Geomagnetic Activity in Human Subjects [32] Heart Rate Variability Reflecting the Dynamics of Solar Activity [33] Solar and geomagnetic activity effects on heart rate variability [34]	Multicenter	2013–2018	Correlations between various weather hazards (solar and geomagnetic activity) on cardiovascular and nervous system	10–703 humans	-
Meta-analyzed heart rate variability, exposure to geomagnetic storms, and the risk of ischemic heart disease [35], which is the leading cause of death worldwide [37,38] Cardiac atrophy after bed rest and spaceflight [39] Mechanism of spaceflight-induced changes in left ventricular mass [40] heart rate and daily physical activity with long-duration habitation of the International Space Station [41] Effect of microgravity on cardiac shape: comparison of pre- and in-flight data in mathematical modeling [42]	Multicenter	1997–2014	Morphological cardiovascular changes in space flight conditions	7–49 astronauts	>9 days
Risk of Cardiac Rhythm Problems during Spaceflight [43] Biomedical Results of Apollo [44] Biomedical results from Skylab [45] Effects of Prolonged Spaceflight on Atrial Size, Atrial Electrophysiology, and Risk of Atrial Fibrillation [46] Effect of short- and long-duration spaceflight on QTc intervals in healthy astronauts [47]	Multicenter	1975–2018	Spaceflight related alteration of cardiac electrophysiology Cardiac rhythm disturbances during space missions	7–33 astronauts	5 days–6 months
Fluid shifts, vasodilatation and ambulatory blood pressure reduction during long-duration spaceflight [48]	Multicenter	2015	The shift of blood and fluids from the lower to the upper body, as well as the effects on arterial pressures on the ISS	13 astronauts	192 days
Increased postflight carotid artery stiffness and inflight insulin resistance resulting from 6-mo spaceflight in male and female astronauts [49]	Multicenter	2016	Post-flight arterial stiffness in the upper region of the body; development of insulin resistance	8 astronauts	6 months
Carotid and Femoral Artery Intima-Media Thickness During 6 Months of Spaceflight [50]	Médecine Physiologie Spatiale Université, France	2016	The effects of microgravity exposure on conduit artery diameter and wall thickness	10 astronauts	6 months
Assessment of Jugular Venous Blood Flow Stasis and Thrombosis During Spaceflight [51]	Multicenter	2019	Risk of impaired cerebral venous outflow and jugular venous thrombosis in long-duration spaceflight	11 astronauts	Long durations (not specified)

ISS—International Space Station.

The circadian rhythm is an important factor to consider in space travel. Increased workload, variations in gravitational force and disturbances of the light–dark cycles elevate the risk for abnormal circadian rhythms, a known risk to cardiovascular health [36]. While currently there is no evidence suggesting a link between space radiation and disrupted circadian rhythms in humans, microarray analysis in CB6F1/Hsd female mice exposed to different types of radiation, including gamma (γ-IR ^137^Cs; 0.4–1.6 Gy, 0.662 MeV) and HZE ions (^14^Si-IR; 0.04–0.32 Gy, 260 MeV/n and ^22^Ti-IR; 0.03–0.26 Gy, 1 GeV/n), revealed five differentially expressed genes linked to the regulation of circadian rhythms 16 months post-exposure: Per2, Per3, Cry2, Bhlhe41, and Arntl [52]. The cardiovascular significance of Per2 lies in its cardioprotective properties during ischemic events [53], influencing processes such as fatty acid metabolism, endothelial dysfunction, and cardiac arrhythmias when disrupted [54]. Additionally, the involvement of Per3 in cardiac regulation and its associated link with obesity and high body mass index demonstrates the sensitivity of the circadian rhythm feedback loop to pathway changes [55].

Refs.[56,57,58,59,60,61,62,63] Alterations within cardiomyocytes have also been observed in cardiac cells exposed to either radiation or microgravity conditions, including increased DNA breaks [23], enhanced transcription of DNA repair genes [26,64], upregulation of Yes-associated protein (YAP1), which plays a role in cardiac proliferation [26,27], ANP and BNP expression that promote cardiac remodeling [24], an increase in inducible nitric oxide synthase (iNOS) transcription and NO [25], impaired K^+^ signaling, which comes with an increased risk for developing arrhythmias [65], and cardiac atrophy [66,67]. Notably, these damaging effects may intensify when microgravity and radiation are combined, thus creating additive or synergistic effects [26]. Investigating the interaction between radiation and microgravity exerting their effects on cells would greatly enhance our understanding of the space environment.

### 2.3. Effect of Radiation on the Vascular System

Other essential features of radiation-induced cardiovascular disease are the macro- and microvascular consequences of high-dose ionizing radiation. In particular, possible macrovascular complications include an increased risk of atherosclerosis and myocardial infarction [68]. Due to its especially potent effect, Yu et al. examined ^56^Fe ion radiation effects on the aortas of male apolipoprotein E-deficient (apoE(-/-)) mice and observed accelerated atherosclerotic development. They noted the aortic root to be exceptionally sensitive post-irradiation [69]. Additional studies have shown that radiation damage induces an influx of granulocyte-predominant inflammatory cells leading to macrophage-rich, inflammatory lesions susceptible to intraplaque hemorrhage [70].

On the microvascular scale, radiation induces an aged phenotype of the endothelium. A recent study differentiated the effects of space radiation from those of microgravity on the endothelium [71]. Space radiation was associated with hypoxia, inflammation, DNA repair and apoptosis. Supporting the activation of the inflammasome is the study of Shampa Chaterjee et al. [72]. This study showed that gamma or proton rays at doses seen with space flight increase oxidant enzymes, ICAM-1 and NLRP3. These prove increased inflammation. Another study focusing on the blood–brain barrier revealed an increase of 8-oxo-deoxyguanosine after exposure of the previously mentioned ^56^Fe ion radiation [73]. This effect persisted for the endothelium as long as 7 days after exposure. During this period, the zonula occludens 1 proteins were less expressed, with a corresponding PECAM1 increase. This means both enhanced permeability due to leaky zonula occludens between endothelial cells and potential inflammation following radiation.

The principal mechanisms contributing to the development of radiation-induced vascular diseases involve oxidative stress, apoptosis, inflammation and DNA methylation (Table 3) [74,75,76,77,78]. Excessive reactive oxygen species (ROS) are generated in the mitochondrial respiratory chain during stress-induced oxidative metabolism. These unstable molecules can damage DNA, RNA, and proteins, ultimately leading to cell death [79]. Additionally, high levels of ROS induce endothelial dysregulation of the vessels, as evidenced by increased xanthine oxidase (XO) activity and decreased nitric oxide (NO) production. In particular, XO upregulation has been linked to elevated arterial stiffness in Wistar male rats which is an indicator of cardiovascular disease [80].

While current data indicate that various biomarker modifications, such as increased levels of oxidative stress levels, inflammation and DNA damage during spaceflight have been linked to cardiovascular disease development on Earth, a causal link with cardiovascular adaptations during spaceflight has not yet been established [81,82]. Limited data on cardiovascular disease (CVD) biomarkers throughout the astronaut lifespan has been published, primarily focusing on traditional biomarkers, like cholesterol levels and glucose metabolism variables, during or near flight. Continued surveillance of cardiovascular biomarkers and long-term health outcomes are required to define health risks [83].

**Table 3:** Mechanisms of radiation-induced endothelial dysfunction and mechanisms of HZE particles for vascular damage.

Radiation Injury	Oxidative Stress	Metabolic Dysfunction	Apoptosis	Fibrosis
Mechanism of endothelial dysfunction	Damage to mitochondrial proteins, followed by impairment of mitochondrial electron transport chain [76]	Inactivation of PPAR-alpha with subsequent atherosclerosis [74]	DNA breaks [23]	Cytokine-mediated release of fibrotic factors [78]
Processes related to HZE particles leading to vascular damage	DNA methylation resulting in tissular degeneration and accelerated atherosclerosis [84]	Decreased angiogenesis [75]	Increased ROS generation with subsequent NO and endothelial relaxation impairment [77]	

HZE—high atomic number and energy.

### 2.4. Prevention against Radiation Injuries

**Figure 2:**
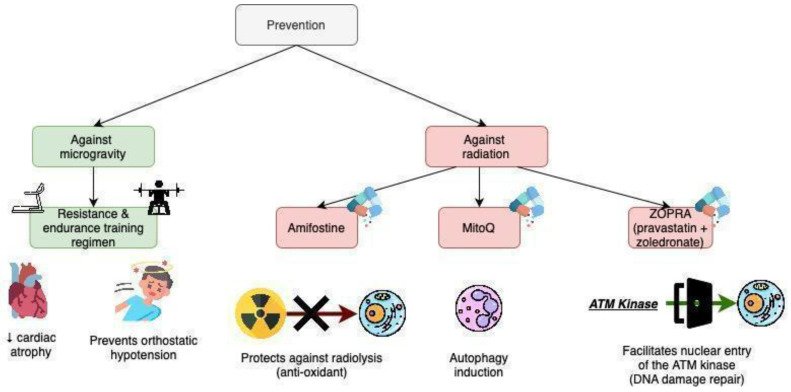
Prevention strategies for mitigating cardiovascular impacts of microgravity and space radiation exposure in astronauts [56,57,61,62,63,72,85,86,87,88,89,90,91,92]. The arrow pointing down indicates a decrease (e.g. decrease in cardiac atrophy).

Recent advancements in radioprotective drugs offer promising new methods for mitigating the harmful effects of radiation exposure in the space travel context (Figure 2). There are a number of drugs shown to be useful in preventing radiation-induced damage. Gamma-Tocotrienol (GT3) is a natural, non-toxic vitamin E isoform that possesses enhanced anti-oxidant and radioprotective properties [85]. GT3 levels in endothelial cells are at least 25 times higher than α-tocopherol, the most common form of vitamin E [85]. Moreover, GT3 is a 3-hydroxy-3-methyl-glutaryl-coenzyme A (HMG-CoA) reductase inhibitor participating in the regulation of cholesterol production as well as in the synthesis of peroxy-nitrite, a powerful oxidant associated with endothelial dysfunction [86,87]. Several studies highlight the radioprotective benefits of GT3 administration. In human umbilical vein endothelial cells (HUVECs), which were exposed to 0.2–5 Gy of ^137^Cs, the treatment with 0–5 μM of GT3 resulted in reduced double-strand breaks and chromosomal aberrations formation [88]. GT3 is also involved in the regulation of genes related to cellular response and recovery processes after exposure to ionizing radiation, such as oxidative stress, regulation of cellular death and angiogenesis in HUVECs [85]. Therefore, GT3 could serve as a potent radiation countermeasure alternative to conventional radioprotective compounds.

Another protective agent against radiation is MitoQ, a mitochondria-targeted antioxidant that induces autophagy in normal cells by inhibiting mitochondrial respiration [56,61,62]. Other approaches to radioprotection include enhancing DNA repair processes. The combination of pravastatin and zoledronate (called ZOPRA) can promote the entry of ATM kinase into the nuclei, which can trigger repair processes for DNA damage [57,89,90]. Amifostine, a chemoprotective agent, decreases DNA damage through its anti-oxidative method of action and protects against radiolysis [91,92]. However, its use is limited due to its side effects, such as severe hypotension, nausea and vomiting [92]. The last compound, LGM2605, is the synthetic compound of secoisolariciresinol diglucoside. The latter is found in whole-grain flaxseed. LGM2605 is a scavenger of radiation-induced ROS, reducing the ionizing species arising from radiation [63,72]. It inhibits proinflammatory signaling pathways, thus inhibiting the expression of NLRP3 and ICAM-1 [72]. These treatments have yet to be implemented in space-flight protocols. Thus, the side-effects are yet to be considered as well as their long-term feasibility.

## 3. How Does Microgravity Affect the Cardiovascular System?

### 3.1. Fluid Redistribution and Loss

On Earth, the 1 g force of gravity imposes a hydrostatic gradient on a person’s body, with a caudal mean arterial pressure of ~200 mmHg and a cephalic pressure of ~70 mmHg [93]. Consequently, about 75% of the blood volume is found in the venous system, primarily due to the vessels’ greater compliance compared to arteries [94]. Upon entering the microgravity environment experienced during spaceflight, the loss of hydrostatic gradient results in a redistribution of fluids within the cardiovascular system. A uniform mean arterial pressure (~100 mmHg) is now present [95]. The two liters of blood volume typically pooled in the lower extremities is shifted upwards to the head [39]. Additionally, there is a loss of plasma volume, which occurs when entering the microgravity environment due to reduced water intake.

#### Spaceflight Associated Neuro-Ocular Syndrome (SANS)

Spaceflight Associated Neuro-ocular Syndrome (SANS) was observed in astronauts. Its probable pathophysiology is related to a reduction in gravity-induced venous drainage of the brain and cephalad fluid shift in microgravity. A correlation between resting blood pressure and SANS incidence was observed in astronauts, as well as different-sex incidence [96].

Recent findings show that intracranial pressure is slightly elevated in microgravity [97,98]. Visual impairments to astronauts are considered as one of the greatest health risks for space travel [97,99,100]. It has been hypothesized that the syndrome is triggered by a decrease in the pressure gradient between the eye and the brain, as intraocular and intracranial pressure are disproportionately adapted in space [97]. Moreover, a change in electrocortical activity has been reported above the occipital and sensorimotor regions in space, resulting in an increase in alpha waves in the EEG [101,102].

### 3.2. How Does Microgravity Impact the Heart?

#### 3.2.1. Microgravity and Its Effect on the Heart’s Resting and Exercise Physiology

In the first few days of a flight, cardiovascular output (CO) increases by almost 35%. This is explained by an increase in the stroke volume and reflex bradycardia. From this point, the CO slightly falls. It further increases in a few weeks as the already high stroke volume is enhanced by an augmentation of the heart rate (HR) [41,42].

The main impact space flight has on the heart’s exercise physiology is the HR. ISS astronauts have lower post-flight peak HR than pre-flight peak HR. The unfavorable change is similar to the change in peak VO2. Peak VO2 of ISS astronauts diminishes by 17% in the first few days in comparison with pre-flight peak VO2. Although it recovers during the upcoming weeks, the peak VO2 post-flight remains below the pre-flight peak VO2 by 15%. The silver lining is that the speed of cardio-vascular adaptations is inversely correlated with the amount of exercise done in space [43,44].

#### 3.2.2. Microgravity and the Heart’s Geometry

Microgravity has deleterious effects on the heart’s geometry and function (Figure 1), including a reduction in ventricular size and cardiac atrophy. For instance, astronauts experienced a 12% decrease in left ventricular mass after just a 10-day spaceflight [39]. Cardiac atrophy is partly caused by the lower metabolic demand on the heart in the weightless environment of microgravity, where movement requires less effort compared to Earth [40,41]. In addition to atrophy, the heart transitions from its usual elliptical shape on Earth to a more spherical shape in microgravity, which compromises its efficiency [42]. Despite astronauts’ superior cardiovascular fitness compared to the average person, these changes in heart shape still take a toll on their cardiovascular health.

#### 3.2.3. Microgravity and Arrhythmias

Cardiac arrhythmias play an important role in morbidity and mortality in different pathological and physiological situations [103,104]. Cardiac arrhythmias have also been observed during spaceflight, with microgravity being a precipitating factor. Although weightlessness solely has not been directly linked to arrhythmias, which are believed to be related to underlying heart disease, they remain a serious concern for space agencies [43]. The risk of astronaut incapacitation during critical moments, coupled with the challenges of performing resuscitation in space, underscores the importance of addressing this issue. Explicitly, premature ventricular complexes (PVCs), ventricular tachycardias (VTs) and premature atrial complexes (PACs) are common in microgravity and have been documented during the Apollo flights, the Skylab studies and the Mir program [44,45,105].

Apollo 15 was the first manned space mission where arrhythmias other than PVCs have been observed. Two periods of 5 bigeminal PVCs and 10 PACs have been noted for one Apollo 15 astronaut. As a result, procainamide and lidocaine were made available on board in the upcoming missions. The astronaut later proved to have previously undiagnosed coronary artery disease which contributed to an acute myocardial infarction two years later [44]. Additionally, a 14-beat episode of VT with a maximum rate of 215 beats per minute (bpm) has been described in an asymptomatic MIR male astronaut [105].

Other noticeable changes in the ECG were an elevated QRS magnitude in two-thirds of the crew members with remission within 5–10 days after return [45]. The cranial fluid shift due to the microgravity environment increases the venous return resulting in enlarged heart cavities, which could explain the increased QRS magnitude. Additional observations were made with regard to the lengthening of the PR interval due to an increased vagal tone. Reinforcing this finding, the PR length during in-flight exercise was similar to the PR interval evaluated in pre-flight conditions [45].

Results from the Skylab missions reveal a plethora of abnormal electrocardiographic findings, with ventricular ectopic beats being once more the most frequent arrhythmia. Ventricular ectopic beats appeared both sporadically, as experienced by the Skylab 4 crew, and during strenuous physical activity, such as high levels of deconditioning countermeasure exercises and extravehicular activity (EVA) [45]. EVAs are arduous and stressful operations that are closely monitored with an ECG to ensure the astronauts’ safety. One member had 80 isolated premature ventricular beats of unifocal origin during 6.5 h of observation. These ectopic ventricular beats did not lead to an alteration of the hemodynamic function or to signs of myocardial ischemia. The astronaut had intermittent episodes of atrioventricular junctional rhythm occurring during rest throughout the flight [45].

Another arrhythmia concern during space travel is atrial fibrillation (AF), a prevalent type of cardiac arrhythmia within the general population, impacting more than 30 million people globally [106]. AF implies a higher risk of medical complications, including stroke, dementia, heart failure and cardiovascular disease, thus increasing the mortality rate by up to 90% [106]. AF has been a documented condition within the space travel crew with 17 evidenced instances of the disease among 317 astronauts, having a prevalence of 5%. Although the peak prevalence of AF is 17.8% in individuals above the age of 85 [107], astronauts exhibit a significantly younger presentation, as all affected active astronauts are aged between 41 and 45 years [46].

The corrected QT interval (QTc) prolongation is extremely common. Specifically, in short-duration flights, 24% of the ISS astronauts experience QTc prolongation, while long-term flights come back to an astonishing 66% of subjects sustaining QTc prolongation. Prolongation of the corrected QT interval (QTc) over 0.45 seconds has been reported in 24% of the MIR mission’s crew, as well as during long-term spaceflights, but not in short-term missions [47]. Several factors may contribute to this instance. Firstly, bradycardia has been associated with long-duration space travel which could contribute to the occurrence of LQTS [47]. Moreover, prolongation of QT is documented as a side effect of certain medications, such as ciprofloxacin, haloperidol, phenergan and macrolides (e.g., azithromycin), which have been administered to the astronauts [105].

The specific etiology of arrhythmias is unknown. However, there are five proposed mechanisms: (1) hypercarbia—the lack of functional fans, as well as the ten times higher CO_2_ in the atmosphere, contribute to arrhythmias, (2) higher than normal levels of catecholamines and stress hormones, (3) electrolyte disturbance due to poor nutrition, (4) chronic undiagnosed atherosclerosis, (5) drugs influencing ECG parameters [43,108]. The electrolyte disturbance (hypokalemia) issue was overcome in the Apollo missions following Apollo 15 by adopting a high potassium diet, improving fluid balance and lowering fatigue levels. This proved beneficial, as no significant arrhythmias were seen in the following Apollo missions [44].

There are limited studies on the long-term effects of microgravity. Studies of more than six months after the space flight are not so conclusive [109]. Moreover, more information-rich data comes from animal models rather than human models [110].

### 3.3. How Does Microgravity Impact the Vessels?

Vascular functional and morphological changes occur during spaceflight as microgravity can alter smooth muscle contractility [51,111,112,113,114,115,116,117,118]. As a result of leg volume reduction due to the microgravity-induced fluid shift and skeletal muscle atrophy in prolonged microgravity, a reduction of peripheral vascular resistance occurs, particularly in the lower extremities, resulting in an increased blood flow. This is accompanied by a slight decrease in systolic, diastolic, and mean arterial blood pressures [48]. Furthermore, after a six-month spaceflight, arterial stiffness increases, along with the carotid intima-media thickness [49,50].

Understanding the role of microgravity and radiation in cardiovascular disease risks is challenging because confounding factors, such as lifestyle and genetics, are thought to contribute to these diseases significantly [83]. This complicates epidemiological studies, making it hard to identify meaningful differences from baseline disease rates without a large study population. This challenge is critical in astronaut studies due to their small sample size. Thus, selecting and designing experimental models is essential to ensure that the mechanisms of cardiovascular disease and the study outcomes are clinically relevant and applicable to humans [119].

Furthermore, statistical models and epidemiological strategies failed to provide an unbiased comparison with astronauts who have flown in space when analyzing long-term health outcomes, such as incidence of chronic disease and mortality after exposure to spaceflight hazards [120].

While space medical research has delivered significant contributions to space missions, the field also offers benefits that extend beyond space exploration. Physiological changes in microgravity provide valuable insights into terrestrial diseases. Astronauts on long-duration spaceflights experience conditions like increased insulin resistance, endothelial dysfunction, muscle atrophy and bone density loss [113,121,122,123]. Research into countermeasures, may, thus, improve disease treatments on Earth. Additionally, the harsh conditions of space also provide valuable insights into metabolic changes, protein and nucleic acid crystallization and drug efficacy, which could lead to new drug discoveries [19].

Furthermore, there are future opportunities regarding medical technology. Space exploration has allowed the development of novel real-time remote health monitoring technologies and robotic systems to deliver medical assistance while undergoing space missions. These advancements have concluded in Earth-based applications in intensive care units, where continuous remote patient tracking could be performed. Additionally, the need to prevent bone density loss among astronauts has pushed the discovery of the effects of vibration therapy, which could mimic the effects of exercise on bones, leading to bone and muscle growth. This led to the development of a rehabilitation chair that uses vibrations to simulate weight-bearing exercises and it is now used in clinical settings, especially in sports medicine, to reduce muscle atrophy and maintain joint flexibility after injury or surgery [124].

Although the effects of microgravity and space radiation in space are not directly comparable to conditions on Earth, there are some parallels and insights that can be drawn. The lack of gravitational force exposes astronauts to reduced mechanical loading, resulting in bone remodeling imbalances. These changes are also seen in osteoporosis [125]. However, the causes, timelines and approach to this disease are different than those for spaceflight-induced bone density loss. Additionally, research involving long-term bed rest or immobilization on Earth can mimic the effects of microgravity to an extent [126,127,128], as reduced physical activity leads to cardiac atrophy and orthostatic intolerance. Nonetheless, the effects are less severe due to the residual gravity and the presence of gravity-dependent bodily functions, as well as the absence of radiation hazards [127].

The renin–angiotensin–aldosterone system has been proposed as an underlying mechanism for the hypertrophic changes in vessels seen in microgravity. The concentrations of these hormones are elevated during spaceflight and provide a direct link, since angiotensin II has been shown to promote oxidative stress, inflammation, and collagen deposition in the arterial wall, leading to structural changes that impair arterial function [49,50,129,130].

An important aspect of vascular changes during spaceflight with potential clinical implications is exemplified by Virchow’s triad [131]. All three elements of Virchow’s triad are present in microgravity, increasing the risk of thrombi (Table 4). For instance, an astronaut was diagnosed with an occlusive internal jugular vein thrombus 50 days into an ISS mission, requiring anticoagulant therapy [51]. This highlights the possible risks of venous thrombosis in microgravity, and the potential of more clinical occurrences.

### 3.4. Prevention against Microgravity Effects

Prevention methods should focus on two deleterious effects of microgravity: ROS-related damage and cardiac deconditioning. Propranolol was previously studied for its effectiveness in inhibiting xanthine oxidase and superoxide [80,132]. This impedes the formation of oxygen radicals. Another way to prevent ROS damage from microgravity is to use an appropriate diet [133,134]. Modern packaging and preservation methods have allowed a prolonged food shelf life, thus preserving nutritional value and aiding in maintaining appropriate antioxidant levels among astronauts [134]. Protective measures can be achieved by consuming a diet abundant in natural antioxidants such as polyphenols, carotenoids and vitamins (vitamin C and E) [135,136]. However, we still need more information from space flight reports to establish whether these measures are feasible in the long run.

Cardiac deconditioning is already addressed using aerobic exercise and weight training, as well as trousers that pull blood back into the lower limbs using pressure differences [60,137,138,139]. However, a recent report suggested the use of ballisto-cardiography and seismo-cardiography to monitor cardiac deconditioning [58]. It also proposed the JUMP training regimen, a regimen of high-intensity physical exercise. JUMP has a positive effect on maintaining the VO2 as well as the stroke volume. It was insufficient to combat orthostatic hypotension upon arrival, the authors suggesting a combination of exercise routine with restoration of plasma volume loss days before landing [58,59].

## 4. Limitations of the Study

A significant limitation of our study, which separates the effects of microgravity and radiation on the cardiovascular system, is that it may not fully capture the complex interactions that occur when both stressors are experienced simultaneously. In reality, astronauts are exposed to both microgravity and heightened radiation levels concurrently, and their combined impact may differ from the sum of their individual effects. Focusing on these factors in isolation may overlook potential synergistic effects, such as how microgravity-induced fluid shifts could interact with radiation-induced vascular damage. Therefore, separating these stressors limits the study’s validity, and the conclusions drawn may not accurately reflect the integrated physiological response astronauts experience during space missions. Future research should prioritize examining these factors together to provide a more comprehensive understanding of cardiovascular risks in space.

## 5. Conclusions

Space travel presents unique challenges to the cardiovascular system The microgravity environment, along with exposure to cosmic radiation, induces a range of changes, including alterations in HRV, blood flow, and heart geometry. Deleterious effects on cardiac function may manifest at times during spaceflight, as in the case of arrhythmias and orthostatic hypotension.

Changes also occur at cellular and molecular levels with impaired intracellular signaling, inflammation and DNA damage, which may affect cellular function. Understanding the molecular and physiological responses of the cardiovascular system to these extreme spaceflight conditions is necessary for developing effective countermeasures and ensuring the safety of astronauts on future missions. New approaches, such as mitoQ or GT3, could represent therapeutic solutions to the hazards of radiation exposure, while various physical exercise strategies can be effective in preventing cardiac deconditioning during microgravity exposure.

Finally, space travel research offers a promising avenue to broaden the implications for medical science on Earth, while driving innovation in medical technologies.

## Figures and Tables

**Table 1:** Radiation types and radiation exposure effects.

Radiation types		
Radiation type	When does the exposure occur on the ISS?	
Galactic cosmic rays [11]	Higher exposure during the solar minimum [11]	
Solar cosmic rays [13]	During solar storms [13]	
Van Allen radiation belts [11]	Daily, with the passage of ISS through the Southern Atlantic Anomaly Its intensity is higher at the solar maximum [11]	
**Radiation exposure effects**		
**Radiation effect**	**Definition**	**Example in space (likelihood of occurrence)**
Radiosensitivity/tissue reaction [12]	Predisposition towards adverse tissue effects caused by cell death (burns, dermatitis) [12]	Acute radiation syndrome during exceptional solar events (theoretical)
Radio-susceptibility [12]	Predisposition towards cancer development due to cellular modifications [12]	Lung and liver cancer
Radio-degeneration [12]	Predisposition towards radiation-induced aging due to DNA damage [12]	Bone loss and early-onset cardiovascular disease (likely)

**Table 4:** Virchow triad elements in space. Despite the fact that each of the Virchow’s triad elements, consisting of stasis, hypercoagulability and endothelial dysfunction, are susceptible to exacerbations in space-flight conditions, the risk of VTE within astronauts remains unknown [45]. Additionally, constituents of the triad, such as endothelial dysfunction, could be the subject of a synergistic interaction between both stressors. i.e., microgravity and radiation, particularly in the context of longer deep space missions where radiation levels will be notably elevated.

Virchow Triad Element	Alteration in Space
Stasis	Increased venous cross-sectional area Increased pressure gradient Retrograde flow [68,73,78,79]
Hypercoagulability	Enhanced Enhanced fibrinogen levels following spaceflight [71,72]
Endothelial damage/dysfunction (induced via cyclic venous distention—cardiovascular disease)	Increases the expression of vWF, tissue factor Promotes platelet adhesion/aggregation [80,85]

VTE—venous thromboembolism; vWF—von Willebrand Factor.

## Abbreviations

HZE—high (H) atomic number (Z) and energy (E), AF—atrial fibrillation, LF—low frequency, ISS—International Space Station, HF—high frequency, HR—heart rhythm, GT3—Gamma-Tocotrienol, PVC—premature ventricular complex, PAC—premature atrial complex, VT—ventricular tachycardia, LQTS—long QT syndrome.
